# Cancer and Inflammation

**DOI:** 10.1038/sj.bjc.6602370

**Published:** 2005-02-22

**Authors:** A G Dalgleish

**Affiliations:** 1 St George's Hospital Medical School, London, UK

This volume is one of a series under the overall heading of ‘*Progress in Inflammation Research*’, this volume focusing on cancer and inflammation. When I was a medical student, I was very struck by the fact that chimney sweeps got cancer of the scrotum and that some males with braces developed breast cancer. Clearly, this has something to do with chronic irritation, if not inflammation. In pathology lessons, reference was made to cancer being a very heterogeneous disease with many different phenotypes, although I remember reference to Virkhoff's comment that ‘cancer is a wound that doesn't cure’. Once on the wards, I was also struck by the fact that people with chronic ulcerative colitis are 30- to 50-fold more likely to develop cancer of the colon than patients without ulcerative colitis. Bizarrely, we were taught that there was no increased risk with another chronic inflammatory disease, called Crohn's disease, which we now know is far from the truth. It is therefore surprising that the link between chronic irritation and inflammation and the subsequent development of cancer has not been given more prominence or recognised as a potential occums' razor to explain the many different manifestations of cancer.

More recently, many authors have noted the association between chronic infections and cancer and noted that they may well be causally linked. Obvious examples include the induction of hepatitis and hepatocellularcarcinoma by chronic infections of hepatitis B virus and hepatitis C virus, as well as associations between chronic bacterial infections, such as *Helicobacter pylori* and stomach cancer. In this regard, Schistosomiasis has been a recognised precursor of bladder cancer for many years.

This book explains all the various relationships between chronic infection and inflammation and common tumours, focusing on reflux oesophagitis and Barratt's oesophagus and the association with oesophageal cancer, asbestosis and mesothelioma, chronic pancreatitis and pancreatic adenocarcinoma, in addition to other infective agents, for example, human papilloma virus and cervical cancer.

The first scientific chapter deals with cancer as a chronic inflammatory disease and the role of immunotherapy. This is an extremely informative chapter as cancer arises in an inflammatory state but can be controlled by the appropriate induction of an immune response, which itself requires a proinflammatory cytokine production. This chapter deals with the fact that there are several different types of stimulus given at different times and this effects the final programming of T cells. For example, a danger signal for a specific antigen, costimulation, polarisation, localisation, termination, continuation and healing process are all required to induce an effective and appropriate immune response. It is of note that in a chronic inflammatory process that there is active immune suppression of cell-mediated immunity, which of course is required to contain any tumour progression.

The next chapter looks at the very complicated network of inflammatory chemokines and their role in tumour growth and progression to help understand how they may lead to tumour progression and how they may be manipulated to enhance an immune response. It would certainly appear that they are involved in tumour growth, vascularity and metastases and hence represent potential therapeutic targets.

For a tumour to grow, evade and metastasis it has to be able to dissolve normal tissues and this usually involves matrix metallo proteinases (MMMPs) that are produced in inflammatory conditions. There is a very delicate interaction and balancing act between MMPs, inflammation and the development of cancer. Certainly by the time a cancer has become evasive and metastasised, there is an accepted need to inhibit MMP activity. As such, this has been the target for several pharmaceutical companies, and the failure to develop a significant nontoxic agent eventually led to the demise of British biotech who had ‘hung their hat’, so to speak, on this being the most important target in cancer.

In addition to chronic inflammation being associated with downregulation of local cell-mediated immune response (which is not always confined to the local environment as there is evidence that even small tumours can suppress systemic cell mediated immune responses), chronic inflammation enhances angiogenesis as there is a requirement for increased repair and growth factors resulting in increased vascularity. In a chapter on the interplay between inflammation and tumour angiogenesis, Yang Song and Nakarda explore the delicate balance as well as the problem that tumour-associated macrophages are often a poor prognostic marker producing a variety of cytokines, proteases, growth factors and angiogenic factors, etc (in contrast, tumour infiltrating lymphocytes are usually a good prognostic feature in most tumour types). The role of this inflammation and the various pathways involved, including the induction of adhesion molecules and interaction with cell cycle inhibitors, is examined. This leads nicely onto the fact that an end result of inflammation and proliferation is apoptosis and the delicate balance between pro- and anti-apoptotic genes. Apoptotic resistance is common in inflammation and is obviously a risk factor in cancer and therefore also provides a novel therapeutic approach.

It is important to be aware that there are many different pathways involved in chronic inflammation; however, one of the most consistent ones involves cyclooxygenase and the prostaglandin pathways. Cyclooxygenase-2 (COX-2) is induced essentially by inflammation and as such drives proangiogenic activity and reduces immune responsiveness. It is present in a wide variety of chronic inflammatory states, promalignant states, as well as metastases and therefore represents a very good target. Aspirin, of course, is the simplest and cheapest of these agents and has given us the evidence that reduction of chronic inflammation will reduce the incidence of colorectal cancer in patients with chronic inflammation, which includes adenomas and polyps. There are two chapters on the links of cyclooxygenase and cancer as well as the different approaches taken to inhibit this enzyme in both the prevention and therapy of cancer. The last footnote of this book quotes from a book called ‘*Pattern Recognition*’ by William Gibson, who defines apophonia and the spontaneous perception of connections and meaningfulness in unrelated things.

This book does a brilliant job in putting in a fairly logical order the links and association of chronic inflammation and cancer in such a way that one can only wonder why it has not been used as the template for research and understanding as well as the development of new treatments for cancer before.

My only criticisms of this book are minor. Firstly, having established a broad range of chronic inflammatory states and solid tumours it is a pity that the editors have not asked whether this would apply to all the major conditions. Examples, notable by their absence, include lung cancer, which is always preceded by histological, if not clinical, bronchitis for at least two decades. There is also evidence that both prostate and breast cancer are associated with various degrees of an inflammatory state. It is certainly recognised that inflammatory breast cancer is a poor prognostic feature.

The second criticism is that whereas tremendous detail has been explored with regards to chemokines in a chapter by Fran Balkwell, the interaction between many of the inflammatory factors and the cell signalling pathways have not been explored in more detail as there are certainly some fascinating molecular interactions; for instance, in that they may help explain the contribution of the famous Vogelstein progression from benign dyplasia through to metastatic disease. Nevertheless, the compact size of this volume makes it easy to read in one sitting as well as being a valuable source of reference for anyone with a passing interest in the cause and treatment of cancer.

